# Targeting the E3 ligase NEDD4 as a novel therapeutic strategy for IGF1 signal pathway-driven gastric cancer

**DOI:** 10.1038/s41388-023-02619-4

**Published:** 2023-02-11

**Authors:** Ke Wang, Yanping Yu, Wei Wang, Yu Jiang, Yunlong Li, Xunliang Jiang, Yihuan Qiao, Le Chen, Xinhui Zhao, Jun Liu, Angang Yang, Jipeng Li, Rui Zhang

**Affiliations:** 1 Digestive surgery department of Xijing Hospital, Fourth Military Medical University, 710032 Xi’an, China; 2 State Key Laboratory of Cancer Biology, Department of Biochemistry and Molecular Biology, Fourth Military Medical University, 710032 Xi’an, China; 3 Shaanxi Provincial Tumor Hospital, The Second Ward of Gynecological Tumor, 710032 Xi’an, China; 4 State Key Laboratory of Cancer Biology, Department of Immunology, Fourth Military Medical University, 710032 Xi’an, China; 5 School of Clinical Medicine, Xi’an Medical University, 710032 Xi’an, China; 6 Department of Thyroid and Breast Surgery, Xi’an No.3 Hospital, the Affiliated Hospital of Northwest University, 710018 Xi’an, China; 7 Department of Experimental Surgery, Xijing Hospital, Fourth Military Medical University, 710032 Xi’an, China

**Keywords:** Targeted therapies, TOR signalling

## Abstract

The IGF1 signal pathway is highly activated in some subtype of gastric cancer(GC) that exhibits poor survival and chemotherapy resistance. Although the results of clinical trials of anti-IGF1R monoclonal antibodies and IGF-1R inhibitors have been mostly disappointing in unselected cancer patients, some patients benefit from anti-IGF1R therapy in these failed studies. Therefore, it is necessary to characterize the complex IGF signaling in GC and help refine the strategies targeting the IGF1 pathway. We found that GC cell lines exhibit differential responses to the specific IGF1R inhibitor OSI906. According to the phosphorylation status of Akt upon the OSI906 treatment, we divided the GC cell lines into IGF1R-dependent and IGF1R-independent cells. Both in vitro and in vivo experiments indicate that Dox-induced knockdown of NEDD4 significantly suppresses tumor growth of IGF1R-dependent GC cells and NEDD4 overexpression promotes tumor growth of IGF1R-dependent GC cells. In contrast, the proliferation of IGF1R-independent GC cells is not affected by NEDD4 silencing and overexpression. The rescue experiments show that a PTEN-IRS1 axis is required for NEDD4-mediated regulation of Akt activation and tumor growth in GC cells. Clinically, NEDD4 is expressed higher in IGF1-high GC tissues compared with IGF1-low GC tissues and normal tissues, and the co-high expression of NEDD4 and IGF1 predicts a worse prognosis in GC patients. Taken together, our study demonstrated that NEDD4 specifically promotes proliferation of GC cells dependent on IGF1/IGF1R signaling by antagonizing the protein phosphatase activity of PTEN to IRS1, and targeting NEDD4 may be a promising therapeutic strategy for IGF1 signal pathway-driven gastric cancer.

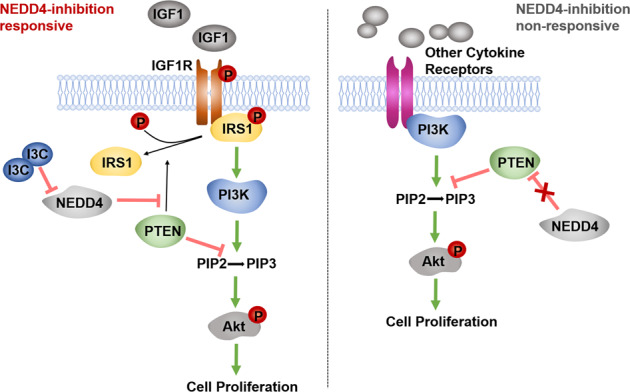

## Introduction

Gastric cancer (GC) is a common and malignant cancer worldwide, of which the incidence rate and mortality rank fifth and third among all types of cancer, respectively [1]. Currently, surgery and adjuvant chemotherapy are still the major therapeutics for GC treatment [2]. So far, the licensed targeted therapies for gastric cancer include trastuzumab (anti-human epidermal growth factor receptor 2, anti-HER2 antibody), ramucirumab (anti-vascular endothelial growth factor 2 receptor antibody), and nivolumab or pembrolizumab (anti-programmed death 1, anti-PD1 antibody) [3]. However, tumor heterogeneity in GC limits the therapeutic effect and drug development of targeted therapies. The rapid development of high-throughput sequencing technology has advanced our understanding of GC biology, and several different molecular classification systems of GC have been proposed in the past decade [4, 5]. Therefore, more studies are needed to explore novel therapeutic targets based on the molecular subtypes of GC and provide more precise treatment for stratified patients.

Recently, two distinct molecular subtypes of GC, mesenchymal phenotype (MP) and epithelial phenotype (EP), have been identified through a genome-wide survey of gene expression data [6]. Interestingly, they found that IGF1/IGF1R pathway is highly activated in the MP subtype, which exhibits poor survival and chemotherapy resistance [6]. Furthermore, MP subtype cancer cells show high sensitivity to inhibition of the IGF1/IGF1R pathway [6], indicating specific therapeutic implications of targeting the IGF1/IGF1R pathway in specific GC patients. IGF1 signaling is an important survival signal for cancer cells as the activator of phosphatidylinositol-3 kinase(PI3K)/Akt signaling [7]. The biological function of IGF1 signaling has been reported in various types of cancer, including hepatocellular carcinoma [8], breast cancer [9, 10], ovarian cancer [11], prostate cancer [12, 13], and colorectal cancer [14]. As for GC, it has been reported that genetic polymorphism of IGF1 is associated with GC risk and prognosis [15, 16]. Although anti-IGF1R monoclonal antibodies and IGF-IR inhibitors have shown potent anticancer effects in preclinical models, clinical trials of these agents have been mostly disappointing in unselected cancer patients. However, some patients benefit from anti-IGF1R therapy in these failed studies, calling for investigations to identify features distinguishing the tumors and host environment of responders from non-responders [17]. Therefore, characterizing the complex IGF signaling in GC will help refine the strategies targeting the IGF pathway.

The Neural precursor cell-expressed developmentally downregulated 4 (NEDD4) functions in tumors mainly as an E3-ubiquitin ligase of tumor suppressor PTEN [18]. A previous report discovered the specific requirement of NEDD4 in the IGF1 surviving pathway [19]. Mechanism research indicated that NEDD4 directly regulates essential mediators in the IGF1 signaling pathway, including the cell-surface expression of IGF1R and the ubiquitination of IRS2 and p-Akt [20–22], which leads us to assume if this vital E3 ligase could be a potential target of the IGF1/IGF1R pathway. Moreover, NEDD4 was reported to enhance IGF1/IGF1R signaling rather than EGF/EGFR signaling by antagonizing the phosphatase activity of PTEN on insulin receptor substrate 1 (IRS1), an essential mediator of the IGF1R/PI3K signaling [23]. PTEN is a widely recognized tumor suppressor gene with high mutation frequency, which exerts the antitumor role mainly through its lipid phosphatase activity against PI3K/Akt signaling pathways [24]. However, PTEN is identified as a dual-specific protein and lipid phosphatase [25]. Several important molecules in tumor biology have been identified as substrates of PTEN protein phosphatase, suggesting a critical role of PTEN in the tumor as a protein phosphatase [26]. Tumor suppressor reactivation has been a long-sought yet elusive therapeutic strategy for human cancer. PTEN is an “obligate haploinsufficient” tumor suppressor gene that functions in a dose-dependent manner, and even a subtle reduction in PTEN level or activity could enhance cancer susceptibility [27], which offers therapeutic opportunities for reactivation of tumor suppressor in cancer treatment. As an E3 ubiquitin ligase of PTEN, NEDD4 controls the protein stability, sub-cellular localization, and enzymatic activity of PTEN [18, 28]. Targeting NEDD4 may be a promising strategy to activate or reactivate PTEN in cancer. Therefore, clarifying the function and mechanism of the NEDD4/PTEN axis in GC is important for defining the therapeutic effects of targeting NEDD4 in IGF signaling-driven GC.

We, therefore, investigated the correlation of NEDD4 and IGF1 signaling in GC and the role of PTEN in this mechanism to define the therapeutic value of targeting NEDD4 in GC. We found that GC cell lines show differential sensitivity to IGF1R targeted inhibitor, and the IGF1/IRS1/IGF1R axis is highly activated in part of GC patients, indicating a stratified approach based on the IGF1/IGF1R activity. Moreover, we found that NEDD4 promotes proliferation and tumor formation of GC cells sensitive to IGF1R inhibitor but not the insensitive ones in a PTEN-dependent manner. Clinically, IGF1R and NEDD4 are highly expressed in GC tissues compared with peritumoral tissues and negatively correlated to the prognosis of GC patients. Taken together, our study demonstrated that NEDD4 could be an ideal therapeutic target for GC patients with abnormal activation of the IGF1/IGF1R pathway, and simultaneous targeting of IGF1R and NEDD4 may be an effective treatment for these patients.

## Results

### Differential responses of GC cell lines to IGF1R inhibitor

**Fig. 1:**
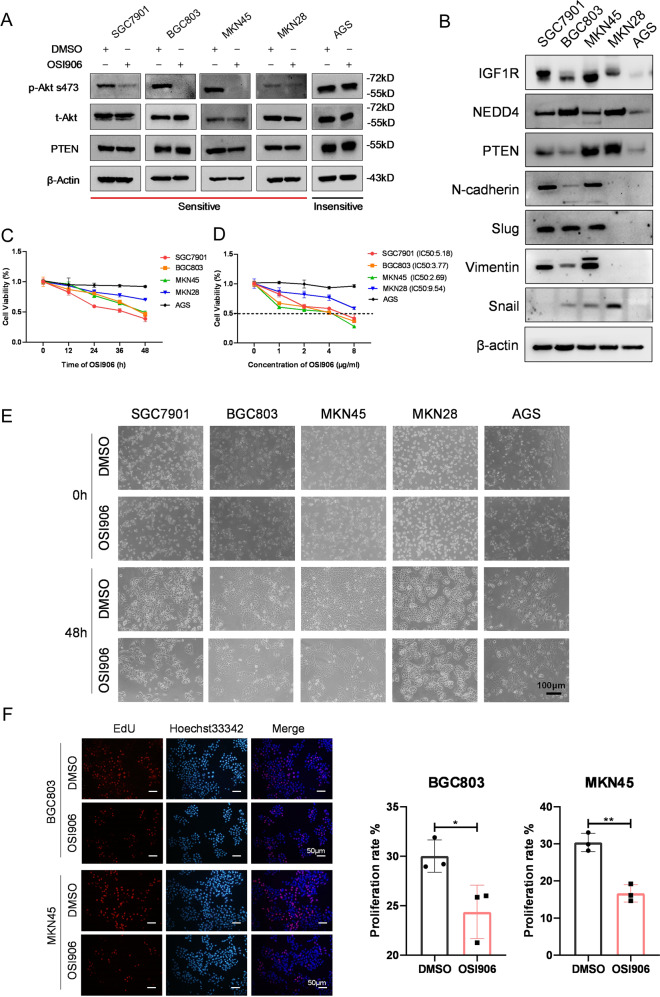
GC cell lines show differential sensitivity to IGF1R specific inhibitor. **A** Western blot analysis of p-Akt s473, t-Akt, PTEN, and β-actin protein levels in five GC cell lines after being treated with DMSO and OSI906 (2 μg/ml, 48 h). **B** Western blot analysis of the constitutive expression of IGF1R, NEDD4, PTEN, N-Cadherin, Slug, Vimentin, and Snail in five GC cell lines. Cell activity (%) detection after treatment of 2 μg/ml OSI906 for 0, 12, 24, 36, and 48 h, respectively (**C**) and treatment of 0, 1, 2, 4, and 8 μg/ml OSI906 for 48 h respectively (**D**) by Cell Counting Kit-8 (CCK-8) assay. The IC50 of SGC7901, BGC803, MKN45, and MKN28 is 5.18, 3.77, 2.69, and 9.54 μg/ml, respectively. **E** Representative Microscope images of five GC cell lines treated with DMSO (0 h and 48 h) and OSI906 (2 μg/ml, 0 and 48 h). **F** The EdU (5-Ethynyl-2′- deoxyuridine) assay showing the proliferation rate of BGC803 and MKN45 after being treated with OSI906 (2 μg/ml) for 48 h. ***p < 0.001, **p < 0.01, *p < 0.05.

IGF1 survival signaling is closely associated with GC occurrence, development, and prognosis. Genomic and proteomic data analysis showed that IGF1/IGF1R pathway is highly activated in a specific MP subtype GC [6]. To determine the role of IGF1 signaling in different GC cell lines, we used a specific IGF1R inhibitor, OSI906. Western blot analysis showed that GC cell lines exhibit distinct sensitivity to IGF1R inhibitor (Fig. 1A, S1A). According to the suppression level of the phosphorylation of Akt, we divided these cell lines into two classes: BGC803, MKN45, SGC7901, MKN28, and BGC823 belonging to the sensitive group, while AGS is the insensitive group. To further validate this finding, we examined the constitutive protein expression of IGF1R, NEDD4, PTEN, and mesenchymal cell markers, including N-Cadherin, Slug, Vimentin, and Snail in five GC cell lines (Fig. 1B). Consistently, IGF1R is highly expressed in cell lines that are sensitive to IGF1R inhibitor. The mesenchymal cell markers are highly expressed in OSI906-sensitive cell lines. Then we investigated the influence of IGF1R inhibitor on the survival and proliferation of GC cells using Cell Counting Kit-8 (CCK-8) assay (Fig. 1C, D) and microscope images (Fig. 1E). The results of CCK-8 assay proved that the IGF1R inhibitor suppresses proliferation of sensitive GC cells in a time- and dose-dependent manner but not the growth of insensitive AGS cells. Moreover, we performed EdU incorporation and Click-iT reaction assay to test the effect of IGF1R inhibitor on the sensitive GC cells and found that proliferation of sensitive cells is obviously suppressed by IGF1R inhibitor (Fig. 1F). Together, these results indicated that GC cell lines exhibit differential responses to IGF1R inhibitor, and the IGF1R inhibitor sensitive cell lines tend to be mesenchymal type.

### An IRS1-PTEN axis is required for proliferation of IGF1R-dependent GC cells

**Fig. 2:**
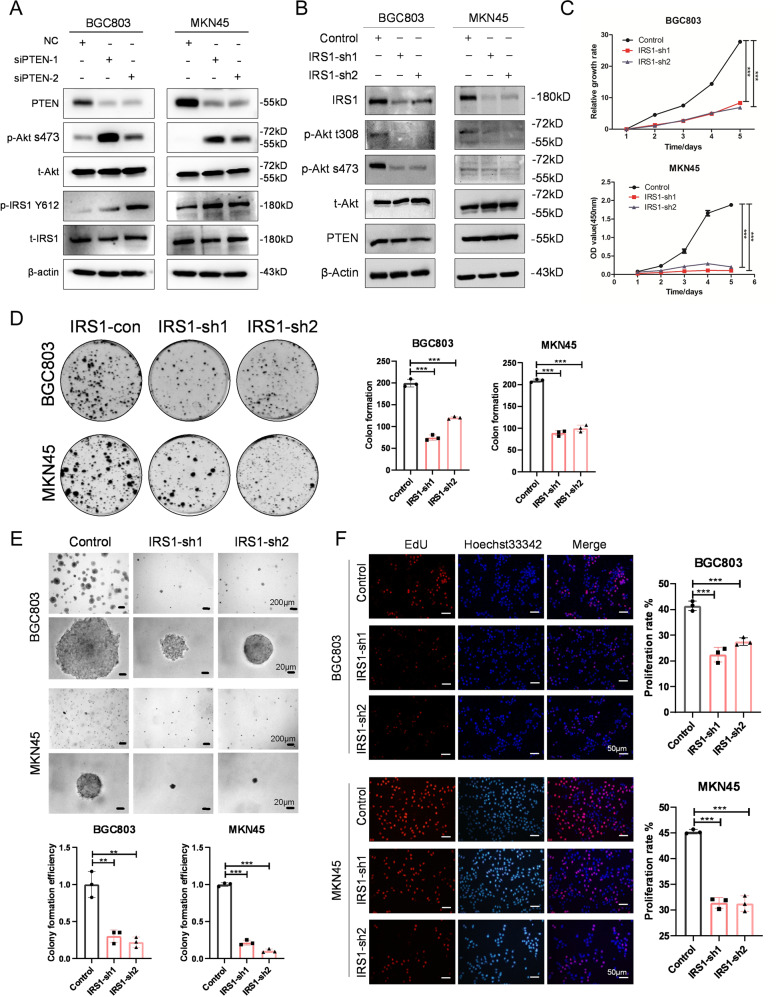
An IRS1-PTEN axis is required for the proliferation of IGF1R-dependent GC cells. **A** Western blot analysis of PTEN, p-Akt s473, t-Akt, p-IRS1 Y612, and t-IRS1 expression in BGC803 and MKN45 cells transfected with PTEN siRNAs. **B** Western blot analysis of IRS1, p-Akt t308, p-Akt s473, t-Akt, and PTEN expression in BGC803 and MKN45 cells infected with lentivirus expressing IRS1-specific shRNA or scramble shRNA. Cell proliferation ability detection of BGC803 and MKN45 cells after IRS1 knockdown by (**C**) CCK-8 assay, (**D**) plate clone formation assay, (**E**) soft agar clone formation assay, and (**F**) EdU assay. ***p < 0.001, **p < 0.01, *p < 0.05.

IRS1, an essential mediator of insulin and IGF1 signaling, has been identified as a substrate of the tumor suppressor PTEN as a protein phosphatase [23]. To verify the role of PTEN as a protein phosphatase of IRS1, we used PTEN-targeted siRNAs. The results of western blotting showed that knockdown of PTEN results in enhanced expression of p-Akt s473 and p-IRS1 Y612 (Fig. 2A). To further investigate the role of the IRS1-PTEN axis in GC cells, we constructed IRS1 stable-knockdown cell lines of BGC803 and MKN45 using two IRS1-specific short hairpin RNAs (shRNAs) by lentivirus infection. The control cells were infected by the lentivirus containing a scramble shRNA. Western blotting confirmed the downregulation of IRS1 and the suppression of phosphorylation of Akt in IGF1R-dependent GC cell lines (Fig. 2B, S1B). To examine the effect of IRS1 knockdown on proliferation of GC cells, we performed CCK-8 assay, plate clone formation assay, soft agar clone formation assay, and 5-ethynyl-2′-deoxyuridine (EdU) assay. The cell viability was significantly weakened upon reduced IRS1 expression (Fig. 2C, S1C). Additionally, IRS1-knockdown cells showed attenuated clone-formation ability (Fig. 2D, S1D) and decreased sphere size and clone-formation efficiency (Fig. 2E) compared with the control cells. Moreover, EdU incorporation and Click-iT reaction assay indicated that knockdown of IRS1 causes a remarkable reduction of cell proliferation rate (Fig. 2F, S1E). Taken together, these data demonstrated that an IRS1-PTEN axis is indispensable for proliferation of IGF1R-dependent GC cells.

### NEDD4 promotes proliferation of GC cells with high activation of IGF1 signaling

**Fig. 3:**
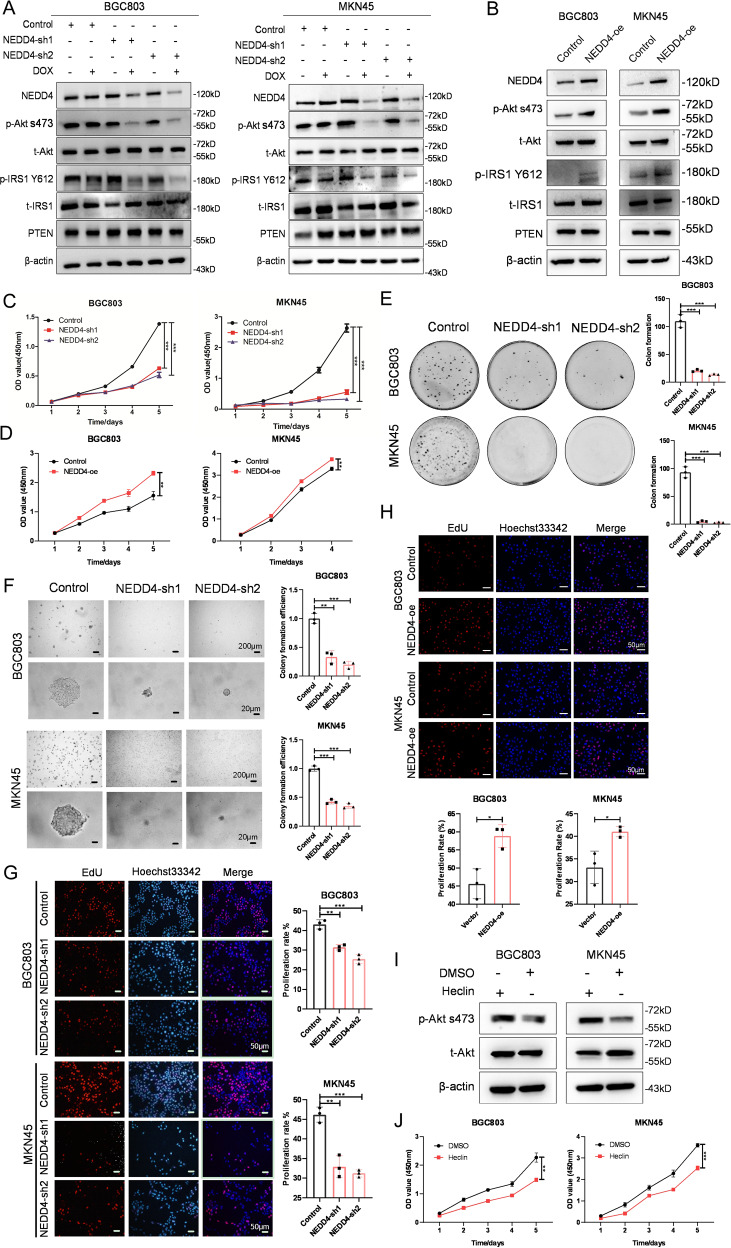
NEDD4 promotes proliferation of IGF1R-dependent GC cells. **A** Western blot analysis of NEDD4, p-Akt s473, t-Akt, p-IRS1 Y612, t-IRS1, and PTEN protein level in BGC803 and MKN45 cells after Dox-induced silencing of NEDD4. NEDD4-con, scramble shRNA; NEDD4-sh1 and sh2, NEDD4 shRNA constructs 1 and 2. **B** Western blot analysis of NEDD4, p-Akt s473, t-Akt, p-IRS1 Y612, t-IRS1, and PTEN protein level in BGC803 and MKN45 cells after NEDD4 overexpression. Cell proliferation ability detection of BGC803 and MKN45 cells after NEDD4 knockdown and overexpression by (**C**, **D**) CCK-8 assay, (**E**) plate clone formation assay, (**F**) soft agar clone formation assay, and (**G**, **H**) EdU assay. **I** Western blot analysis of p-Akt s473 and t-Akt protein levels in BGC803 and MKN45 cells after treatment with Heclin (10 μM) for 48 h. **J** Cell viability detection of cells treated with Heclin (10 μM) by CCK-8 assay. ***p < 0.001, **p < 0.01, *p < 0.05, ns > 0.05.

NEDD4 plays an essential role in IGF1 signaling rather than EGF signaling by antagonizing the protein phosphatase activity of PTEN on IRS1 [23]. To further explore the function of NEDD4 in GC cells, we engineered two different shRNA sequences against NEDD4 in a doxycycline (Dox)-inducible manner and transfected them into GC cells by lentivirus infection. Western blotting showed that Dox-induced silence of NEDD4 suppresses the phosphorylation of Akt and IRS1 in cells with high activation of IGF1 signaling, while the level of PTEN protein barely indicates a decrease (Fig. 3A). Conversely, overexpression of NEDD4 leads to upregulation of phosphorylation of Akt and IRS1 in cells with high activation of IGF1 signaling (Fig. 3B). The CCK-8 assay revealed that knockdown of NEDD4 restrains proliferation and overexpression of NEDD4 promotes proliferation of IGF1R-dependent GC cells (Fig. 3C, 3D and S2A). Similar results were obtained from plate clone formation assay, soft agar clone formation assay, and EdU assay. NEDD4-silencing IGF1R-dependent GC cells exhibited less and smaller clone formation (Fig. 3E), decreased sphere size and clone forming efficiency (Fig. 3F), and declination in cell proliferation rate (Fig. 3G, S2B). Whereas, cells with NEED4 overexpression showed increased proliferation rate (Fig. 3H). To further validate the oncogenic role of NEDD4 in IGF1R-dependent GC cells, we used Heclin, a specific inhibitor for HECT ligases including NEDD4 [29]. As shown in Fig. 3I, phosphorylation of Akt was reduced upon Heclin treatment. The CCK-8 assay showed that Heclin inhibits cell viability of BGC803 and MKN45 cell lines (Fig. 3J). Collectively, these results demonstrated that NEDD4 promotes proliferation of GC cells with high activation of IGF1 signaling.

### IRS1 and NEDD4 are not required for proliferation of IGF1R-independent GC cells

**Fig. 4:**
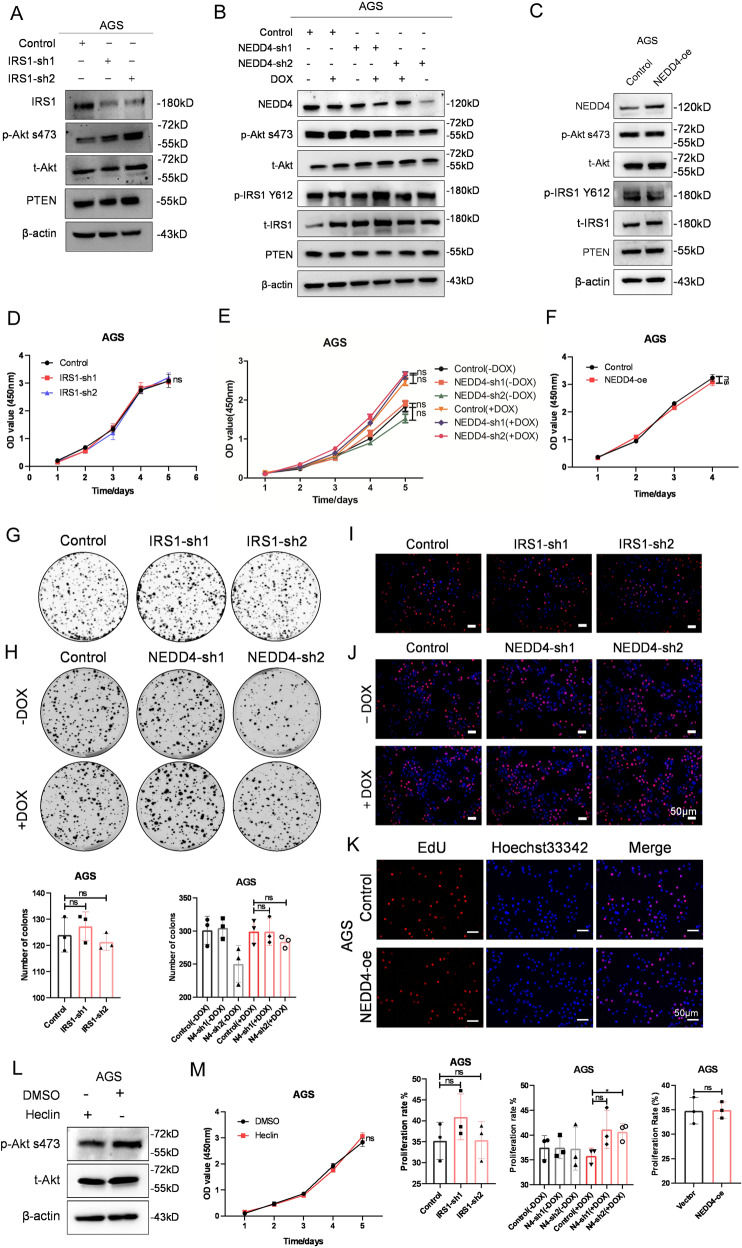
Knockdown of IRS1 and NEDD4 does not affect the proliferation of IGF1R-independent GC cells. **A–C** Western blot analysis of NEDD4, p-Akt s473, t-Akt, p-IRS1 Y612, t-IRS1, and PTEN protein level in AGS cells after silencing of IRS1 and NEDD4 and overexpressing NEDD4. Cell proliferation ability detection of AGS cells after IRS1 and NEDD4 knockdown and NEDD4 overexpression, respectively, by (**D–F**) CCK-8 assay, (**G**, **H**) plate clone formation assay, and (**I–K**) EdU assay. **L** Western blot analysis of p-Akt s473 and t-Akt in AGS cells after treatment of Heclin(10 μM) for 48 h. **M** Cell viability detection of AGS treated with Heclin (10 μM) by CCK-8 assay. ***p < 0.001, **p < 0.01, *p < 0.05, ns > 0.05.

To further validate the role of the IRS1-PTEN axis and NEDD4 in regulating IGF1 signaling, we knocked down IRS1 and NEDD4 and exogenous overexpressed NEDD4 in IGF1R-independent GC cell line AGS. Western blot analysis showed that knowdown of IRS1 and NEDD4 did not suppress the phosphorylation of Akt and IRS1 in AGS cells (Fig. 4A, B), while overexpression of NEDD4 did not lead to increase of phosphorylation of Akt and IRS1 (Fig. 4C). CCK-8 assay indicated that the knockdown of IRS1 and NEDD4, and overexpression of NEDD4 did not affect the cell viability of AGS (Fig. 4 D–F). IRS1 and NEDD4 silencing and exogenous NEDD4 expression in IGF1R-independent GC cell line AGS did not affect the clone formation ability (Fig. 4G, H) and cell proliferation rate (Fig. 4I–K). Moreover, treatment of Hecli did not result in repressed phosphorylation of Akt (Fig. 4L) and decreased cell viability (Fig. 4M).Together, these data indicated that NEDD4 did not promote proliferation of IGF1R-independent GC cells.

### NEDD4 promotes tumorigenesis of GC cells with high activation of IGF1 signaling

**Fig. 5:**
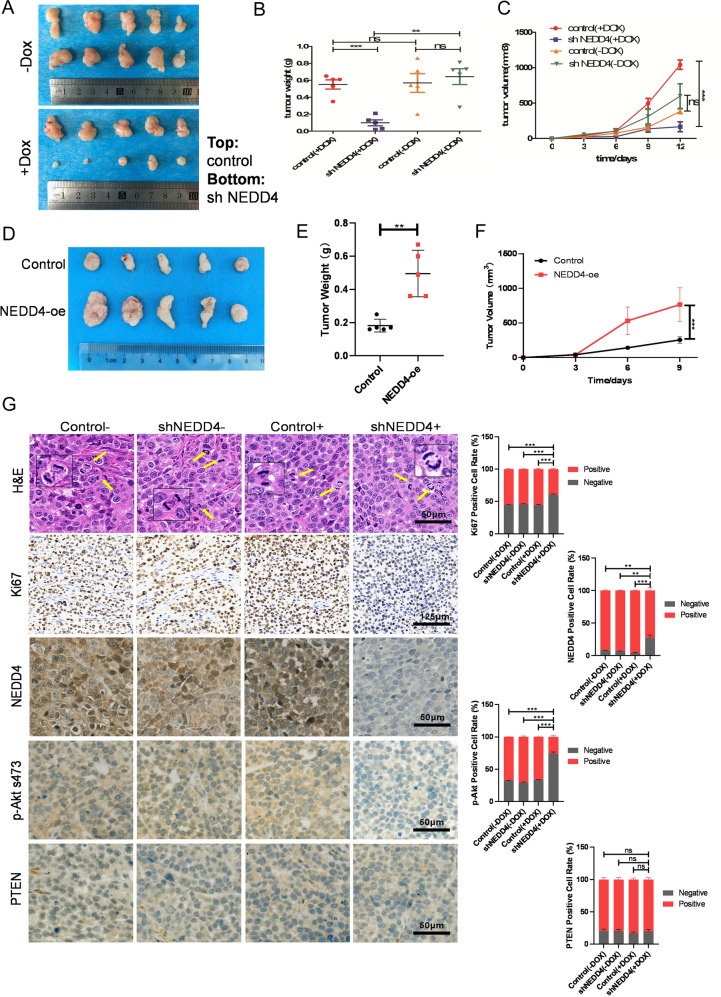
NEDD4 promotes tumor growth of IGF1R-dependent GC cells in vivo. **A** Representative images of tumors in nude mice after the injection of BGC803 cells stably expressing Dox-inducible NEDD4-shRNA and their controls (*n* = 5). **B**, **C** Quantification of tumor weights (**B**) and growth curves (**C**) of xenograft tumors in nude mice. **D** Representative images of tumors in nude mice after the injection of BGC803 cells transfected with NEDD4-expressing plasmids and negative control plasmids (*n* = 5). **E**, **F** Quantification of tumor weights (**E**) and growth curves (**F**) of xenograft tumors in nude mice. **G**, *left* Representative images of the hematoxylin and eosin (H&E) staining, Ki67, NEDD4, p-Akt s473, and PTEN IHC staining in tumor samples. **G**, *right* The percentages of positive cells were calculated by ImageJ IHC Profiler. ***p < 0.001, **p < 0.01, *p < 0.05, ns *p* > 0.05.

To further confirm the function of NEDD4 in GC in vivo, we subcutaneously injected BGC803 cells that stably express NEDD4 targeting shRNA in a Dox-inducible manner and their controls into each flank of nude mice. After injection, we added doxycycline into the drinking water (1 mg/ml) of the +Dox group. We monitored the tumor volumes and plotted the growth curves of the tumors accordingly. Moreover, we isolated the tumors and measured the tumor weights. We found that Dox-induced NEDD4 reduction significantly inhibits gastric tumor growth in vivo (Fig. 5A–C). Moreover, we also established the xenograft tumor model in nude mice using NEDD4-overexpressing BGC803 cells and monitored the tumor volumes and measured the tumor weights (Fig. 5D–F). These results indicated that NEDD4 overexpression promotes tumorigenesis of IGF1R-dependent GC cells. We further detected the expression level of Ki67, NEDD4, p-Akt s473, and PTEN in the tumors using IHC staining. In the +Dox group, konckdown of NEDD4 significantly suppresses cell proliferative activity, as indicated by the percentage of cells positive for Ki67 staining compared with the control group. However, the percentages of cells positive for Ki67 in control (−Dox) and shNEDD4 (−Dox) groups were almost the same as in the control (+Dox) group (Fig. 5G). Moreover, IHC staining confirmed the deletion of NEDD4 and decreased levels of Akt phosphorylation in the shNEDD4(+DOX) group. These in vivo observations confirmed the importance of NEDD4 in proliferation of GC cells with high activation of IGF1 signaling and the potential of targeting NEDD4 for GC treatment.

### The therapeutic benefit of NEDD4-targeting in GC cells is in a PTEN-dependent manner

**Fig. 6:**
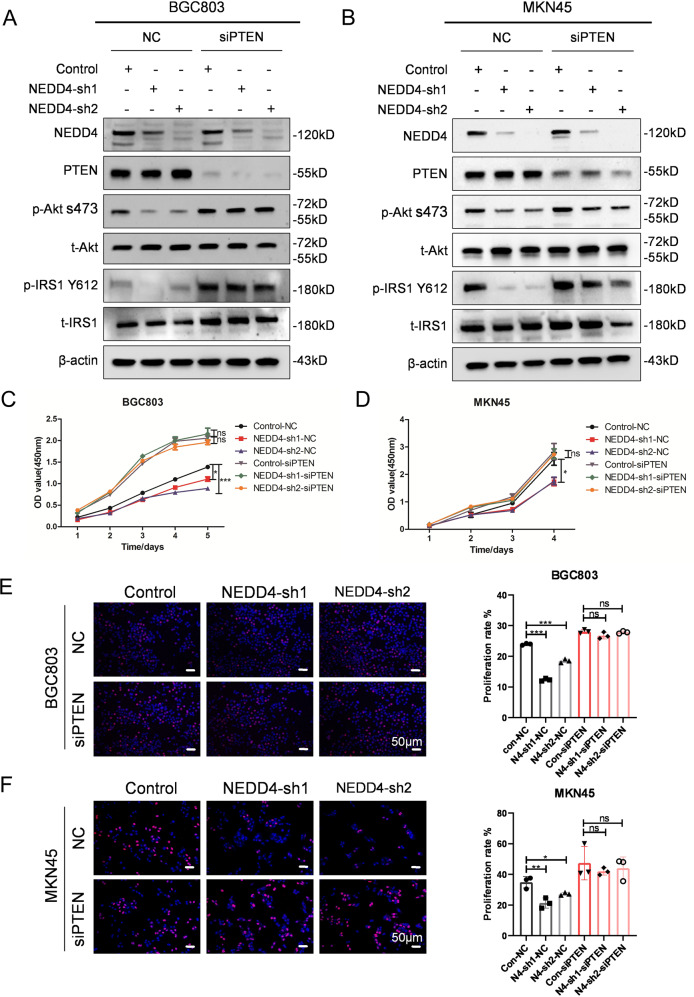
NEDD4 promotes proliferation of IGF1 signaling-dependent GC cells in a PTEN-dependent manner. **A**, **B** Western blot analysis of NEDD4, PTEN, p-Akt s473, t-Akt, p-IRS Y612, and t-IRS1 protein level in BGC803 and MKN45 cells infected with lentivirus expressing scramble shRNA (control), shNEDD4-1 and shNEDD4-2 combined with PTEN siRNA. **C**, **D** CCK-8 assay and (**E**, **F**) EdU assay showing proliferation ability of the indicated cells. ***p < 0.001, **p < 0.01, *p < 0.05, ns *p* > 0.05.

PTEN is a key negative regulator of IGF1 signaling and also an important substrate of NEDD4 [26]. We, therefore, hypothesized that PTEN plays an indispensable role in the regulation of NEDD4 to the IGF1R-dependent GC cells. We performed a rescue experiment by knocking-down PTEN using siRNA in BGC803 and MKN45 cells that stable express NEDD4-shRNAs. As shown in Fig. 6A and B, expression of NEDD4 and PTEN is efficiently decreased, and the inhibition of Akt and IRS1 phosphorylation by NEDD4 silencing is reversed by PTEN downregulation. CCK-8 assay indicated that the knockdown of PTEN improves the cell viability of NEDD4-silencing GC cells (Fig. 6C and D). Similar results were observed in the EdU assay, and the cell proliferation rate of NEDD4-silencing GC cells was upregulated by the reduction of PTEN (Fig. 6E and F). To conclude, PTEN is indispensable in modulating NEDD4 to the proliferation of IGF1R-dependent GC cells.

### The prognostic value of the IGF1/NEDD4/PTEN/IRS1 axis in GC

**Fig. 7:**
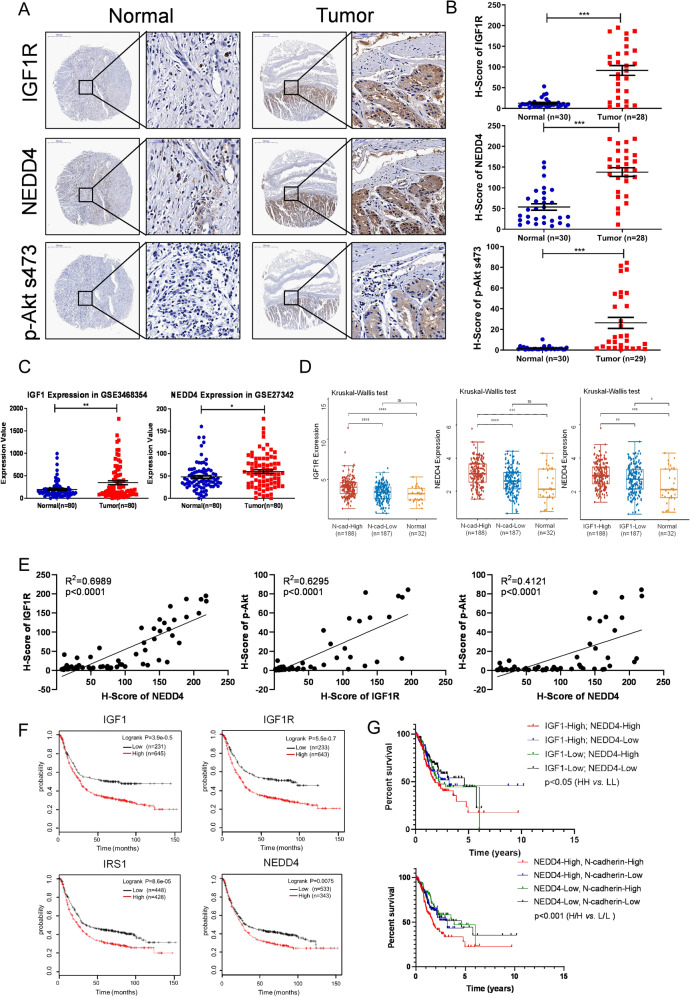
High levels of the IGF1 pathway and NEDD4 are closely associated with poor prognosis of GC. **A** Representative images of IGF1R, NEDD4, and p-Akt s473 expression in adjacent non-tumor tissues and primary GC tissues detected by IHC staining. **B** IHC scores of IGF1R, NEDD4, and p-Akt S473 expressions in adjacent non-tumor tissues and primary GC tissues. **C** Analysis of IGF1 and NEDD4 expressions in normal tissue and GC tissues from Gastric datasets (GSE3468354 and GSE27342, N, normal gastric mucosa, *n* = 80; T, gastric tumor, *n* = 80). **D** Analysis of IGF1R expression in GC stratified by N-cadherin expression and normal tissues, NEDD4 expression in GC stratified by N-cadherin expression and normal tissues, and NEDD4 expression in GC stratified by IGF1 expression and normal tissues in TCGA database. **E** Protein expression correlation of IGF1R, NEDD4, and p-Akt s473 in GC tissues. **F** Kaplan–Meier plots of OS among GC patients with different expressions of IGF1, IGF1R, IRS1, and NEDD4. **G** Overall survival rate of STAD from the TCGA database was analyzed according to the mRNA levels of IGF1 and NEDD4, NEDD4 and N-cadherin. IGF1-High/NEDD4-High (*n* = 103); IGF1-High/NEDD4-Low (*n* = 82); IGF1-Low/NEDD4-High (*n* = 82); IGF1-Low/NEDD4-Low (*n* = 103). NEDD4-High/N-cadherin-High (*n* = 111); NEDD4-High/N-cadherin-Low (*n* = 74); NEDD4-Low/N-cadherin-High (*n* = 74); NEDD4-Low/N-cadherin-Low (*n* = 111). ***p < 0.001, **p < 0.01, *p < 0.05, ns *p* > 0.05.

IGF1 signaling has been found to play an important role in GC [6]. We analyzed the correlation between IGF1 and IRS1 expression and patients’ clinicopathological variables in GC using the TCGA data set. The results indicated that high-level IGF1 and IRS1 expression in GC is significantly associated with a more aggressive tumor phenotype (Tables 1, 2). Expression of NEDD4 is not associated with clinicopathological features but with the prognosis of patients with GC (Table. S1), probably because NEDD4 is overexpressed explicitly in mesenchymal subtype GC. To determine the expression pattern of the IGF1R/NEDD4/Akt axis in GC, we performed IHC staining to detect their expression levels in adjacent non-tumor tissues and primary GC tissues. As shown in Fig. 7A and B, IGF1R, NEDD4, and p-Akt s473 were significantly overexpressed in GC tissues compared with normal tissues. We further investigated the expression pattern of IGF1 and NEDD4 in human gastric cancer cohorts. Consistently, the results showed that IGF1 and NEDD4 were upregulated in GC tissues compared with the normal gastric mucosa (Fig. 7C). And IGF1R was expressed higher in the N-cadherin-high GC tissues than the N-cadherin-low and normal tissues. Expression of NEDD4 was higher in the IGF1-high and N-cadherin-high GC tissues compared with the IGF1-low and N-cadherin-low GC, and normal tissues, respectively (Fig. 7D), indicating the correlation between IGF signaling, NEDD4, and mesenchymal phenotype. We further analyzed the expression correlation of IGF1R, NEDD4, and p-Akt s473 in GC tissues, and the results showed that they are positively correlated with each other (Fig. 7E). Besides, the correlation analysis using the TCGA dataset showed that NEDD4 is positively correlated with the IGF1/IGF1R/IRS1 axis in human GC (Fig. S3A). Moreover, IGF1 positively correlates with mesenchymal phenotype markers (including Snail, Twist1, Zeb1, Slug, Vimentin, MYH11, LEF1, and FAP) and negatively correlated with epithelial phenotype marker MUC1 (Fig. S3B). NEDD4 positively correlates with cell proliferation marker Ki67 and mesenchymal phenotype markers (including Snail, Twist1, Zeb1, Slug, Vimentin, MYH11, LEF1, and FAP), and negatively correlated with epithelial phenotype marker MUC1 (Fig. S3C). To determine the prognostic value of the IGF1/NEDD4/PTEN/IRS1 axis in GC, we analyzed the correlation between IGF1, IGF1R, IRS1, and NEDD4 expression and overall survival (OS), first progression survival (FP), and post-progression survival (PPS) of patients with GC using Kaplan–Meier database. The results indicated that GC patients with high expression of IGF1, IGF1R, IRS1, and NEDD4 show shorter OS, FP, and PPS than those with low expression (Figs. 7F, S3D). Furthermore, we performed a survival analysis of GC using data in the TCGA and found that patients bearing IGF1-high and NEDD4-high tumors have poorer survival (Fig. 7F). It indicates that IGF1 and NEDD4 tend to be co-expressed in GC and indicates a poor prognosis, thus shedding light on the therapeutic benefit of targeting NEDD4 in IGF1 pathway-driven GC. And the co-overexpression of NEDD4 and IGF1 and the co-overexpression of NEDD4 and N-cadherin indicate adverse outcomes in GC patients, suggesting that NEDD4 tends to be highly expressed in mesenchymal subtype GC with increased activation of IGF1/IGF1R signaling pathway and predicts a poor prognosis (Fig. 7D and G). Taken together, these results showed that IGF1 signaling and NEDD4 are overexpressed in GC tissues, especially in the mesenchymal subtype compared with adjacent non-tumor tissues, and are predictors of poor prognosis of GC patients, indicating the clinical value of targeting NEDD4 in treatment of mesenchymal subtype GC with abnormally activated IGF1 pathway.

**Table 1:** Correlations between IGF1 expression and clinical characteristics in patients with GC.

Variables	Expression of IGF1		*p* value
	High (*n* = 188)	Low (*n* = 187)	
*Age (years)*			0.25
Age Mean (SD)	65.2 (10.5)	66.5 (10.8)	
Age Median [Min Max]	66 [39 87]	68 [35 90]	
*Gender*			0.564
Male	124 (65.96%)	117 (62.57%)	
Female	64 (34.06%)	70 (37.43%)	
*Grade of differentiation*			0.001*
G1	3 (1.60%)	7 (3.74%)	
G2	52 (27.66%)	85 (45.45%)	
G3	128 (68.09%)	91 (48.66%)	
GX	5 (2.66%)	4 (2.14%)	
*Tumor invasion*			0.007^*^
T1	2 (0.53%)	4 (2.14%)	
T2	36 (19.15%)	44 (23.53%)	
T3	85 (45.21%)	83 (44.39%)	
T4	58 (30.85%)	42 (22.46%)	
TX	7 (3.72%)	1 (0.53%)	
*Lymph node status*			0.293
N0	47 (25.00%)	64 (34.22%)	
N1	49 (26.06%)	48 (25.67%)	
N2	39 (20.74%)	36 (19.25%)	
N3	42 (22.34%)	32 (17.11%)	
NX	10 (5.32%)	6 (3.21%)	
*Distant metastasis*			0.52
M0	162 (86.17%)	168 (89.84%)	
M1	15 (7.98%)	10 (5.35%)	
MX	11 (5.85%)	9 (4.81%)	
*TNM stages*			0.012^*^
I	15 (7.98%)	38 (20.32%)	
II	56 (29.79%)	9 (29.41%)	
III	82 (43.62%)	68 (36.36%)	
IV	18 (9.57%)	20 (10.70%)	

^*^Statistically significant. *P* value < 0.05 are in bold.

**Table 2:** Correlations between IRS1 expression and clinical characteristics in patients with GC.

Variables	Expression of IRS1		*p* value
	High (*n* = 188)	Low (*n* = 187)	
*Age (years)*			0.375
Age Mean (SD)	65.3 (10.2)	66.3 (11.1)	
Age Median [Min Max]	67 [41 90]	68 [35 90]	
*Gender*			0.564
Male	121 (64.36%)	120 (64.17%)	
Female	67 (35.64%)	67 (35.83%)	
*Grade of differentiation*			0.004*
G1	2 (1.06%)	8 (4.28%)	
G2	57 (30.32%)	80 (42.78%)	
G3	122 (64.89%)	97 (51.87%)	
GX	7 (3.72%)	2 (1.07%)	
*Tumor invasion*			0.012*
T1	2 (1.06%)	17 (9.09%)	
T2	35 (18.62%)	45 (24.06%)	
T3	84 (44.68%)	84 (44.92%)	
T4	61 (32.45%)	39 (20.86%)	
TX	6 (3.19%)	2 (1.07%)	
*Lymph node status*			0.657
N0	47 (25.13%)	64 (34.41%)	
N1	51 (27.27%)	46 (24.73%)	
N2	41 (21.93%)	34 (18.28%)	
N3	39 (20.86%)	35 (18.82%)	
NX	9 (4.81%)	7 (3.76%)	
*Distant metastasis*			0.982
M0	165 (87.77%)	165 (88.24%)	
M1	13 (6.91%)	12 (6.42%)	
MX	10 (5.32%)	10 (5.35%)	
*TNM stages*			0.155
I	20 (11.36%)	33 (18.75%)	
II	52 (29.55%)	59 (33.52%)	
III	83 (47.16%)	67 (38.07%)	
IV	21 (11.93%)	17 (9.66%)	

^*^Statistically significant. *P* value < 0.05 are in bold.

## Discussion

Although the overall survival of GC patients has been increased by optimizing surgical resection and chemotherapy, the stubbornly high incidence rate and mortality still make GC an extremely challenging malignancy worldwide. Advanced progression with multidisciplinary collaboration helps cancer biologists divide the comprehensive molecular classifications of GC. Consequently, it promotes the development of personalized therapy, especially in the molecular targeted therapy strategy [4, 30]. Moreover, as a molecularly and phenotypically highly heterogeneous disease, the progress of targeted therapy for GC is slow. So far, targeted therapies licensed to treat GC include trastuzumab (HER2-positive patients first line), ramucirumab (anti-angiogenic second line), and nivolumab or pembrolizumab (anti-PD-1 third line) [3]. However, clinical trials have shown that these targeted drugs do not significantly prolong survival in patients with GC, and molecular targets’ expression level, response rates, and drug resistance remain crucial barriers to the therapeutic effects of targeted therapies. Therefore, exploring novel therapeutic targets is of great importance for GC treatment.

In this study, we found that IGF1/IGF1R signaling is highly activated in GC, and inhibition of IGF1R potently suppresses the proliferation of gastric cancer cells. IGF1 signaling is a proliferation signal that counts for the survival of tumor cells. Its abnormal activation plays a role in promoting the initiation and progression of various types of tumors. IGF1/IGF1R regulates cell proliferation, angiogenesis, metastasis, metabolism, tumor microenvironment, chemoresistance, stem cells enrichment, and amplification in cancer [31–35]. As for gastric cancer, IGF1/IGF1R promotes tumor growth and metastasis by inducing the expression of Interferon-induced transmembrane protein 2 [36]. NanoString profiling of 64 GCs found that IGF1 is overexpressed in primary tumor deep and matched lymph node metastasis compared with primary tumor superficial, indicating an essential role of IGF1 signaling in regional lymph node metastases of GC [37]. IGF1 signaling is a crucial cancer-promoting pathway that controls cancer development and progression. Previous reports found that high IGF1 level was associated with an increased risk of lung, prostate, breast, and colorectal cancers [38]. Clinically, IGF1 expression is associated with tumor differentiation grade, tumor invasion, and TNM stages in GC, indicating IGF1 pathway may be a promising therapeutic target for GC. Similarly, IRS1 expression is associated with tumor differentiation grade and tumor invasion in patients with GC. Moreover, the genomic and proteomic data analysis found that IGF1/IGF1R pathway is highly activated in one molecular subtype of GC, MP, associated with poor prognosis, markedly low somatic mutation rates, microsatellite instability (MSI), and resistance to standard chemotherapy [6]. Consistently, in the present study, we found that IGF1R is co-overexpressed with N-cadherin in GC, and IGF1 level is positively correlated with mesenchymal phenotype markers (including Snail, Twist1, Zeb1, Slug, Vimentin, MYH11, LEF1, and FAP) and negatively correlated with epithelial phenotype marker MUC1 in GC. Furthermore, IGF1R inhibitor-sensitive GC cell lines highly express mesenchymal phenotype markers, such as N-Cadherin, Slug, Vimentin, and Snail, suggesting that IGF1R-dependent GC cells tend to be mesenchymal subtype. These results indicate that the IGF1 signal is vital in GC progression, especially the MP subtype, which provides the rationale for targeting the IGF1 pathway in GC.

However, outcomes of clinical trials about IGF1R targeted agents have been largely disappointing. Various pathways lead to the resistance to IGF1R-targeted therapy, such as compensatory RTKs activation and alternative integrin signaling [39], and diverse factors dictate the susceptibility of cancer cells to IGF1R targeting, including oncogenic states and cell identities/states [40]. Therefore, clarifying the complex IGF1 signaling pathway in cancer sheds light on therapeutic strategies for directly and indirectly targeting the IGF1 axis. NEDD4-null mice show reduced IGF1 and insulin signaling and mislocalized IGF1R [19]. NEDD4 enhances IGF signaling by mediating the ubiquitination of IRS-2 and IGF1R [21, 41]. In the current study, we found that NEDD4 explicitly promotes proliferation of GC cells with IGF1/IGF1R high activation. Clinically, NEDD4 is higher expressed in IGF1-high GC tissues compared with IGF1-low GC tissues and normal tissues, and the co-high expression of NEDD4 and IGF1 predicts a worse prognosis of patients with GC. Moreover, NEDD4 is co-highly expressed with N-cadherin in GC, and the co-expression of them is associated with adverse outcomes in GC patients. Similar to IGF1, expression of NEDD4 is also positively correlated with mesenchymal phenotype markers (including Snail, Twist1, Zeb1, Slug, Vimentin, MYH11, LEF1, and FAP) and negatively correlated with epithelial phenotype marker MUC1 in GC. However, there is no significant correlation between NEDD4 and clinicopathological characteristics in patients with GC. In comparison, high expression of NEDD4 is correlated with poor prognosis of GC patients. This may be due to the specific overexpression of NEDD4 in mesenchymal subtype GC, which suggests the necessity of stratification according to IGF1/IGF1R expression or mesenchymal/epithelial subtype. Collectively, these results suggest that stratification of GC patients by IGF1 and NEDD4 is necessary, and targeting NEDD4 may be an alternative or combined strategy to address the susceptibility and drug resistance of IGF1 signaling targeted therapy.

PTEN is one of the most frequently mutated tumor-suppressor genes in various tumors. And the role of NEDD4 has been expanded to tumor biology since it was reported to be an E3 ubiquitin ligase of PTEN [18, 42]. However, the role of the NEDD4-PTEN axis in tumor progression remains controversial. Accumulating evidence has shown that NEDD4 functions as an oncogene in a PTEN-dependent or PTEN-independent manner. In bladder cancer, for instance, knockdown of NEDD4 inhibits cell proliferation, migration, and invasion and even induces cell apoptosis by reactivating PTEN [43]. It was also reported that in lung cancer and hepatocellular carcinoma, NEDD4 promotes cancer progression by negatively regulating PTEN [44, 45]. Nevertheless, NEDD4 was shown to promote proliferation of colorectal cancer cells in a PI3K/PTEN/Akt pathway-independent manner [46]. Moreover, the regulation of PTEN by NEDD4 is context-dependent. As an E3 ligase, NEDD4 is found to act as an oncogene by mediating PTEN ubiquitination and degradation [44, 47], whereas NEDD4 can also ubiquitinate and suppress PTEN function without affecting its stability. For example, NEDD4-mediated ubiquitination affects the protein phosphatase activity of PTEN on IRS1 without affecting PTEN protein stability [23]. Previous studies have found that E3 ligases Ret finger protein (RFP) and WW domain containing E3 Ub-protein ligase 1 (WWP1) mediated K27-linked ubiquitination of PTEN, which inhibits antitumor activity of PTEN without affecting its protein level [48, 49]. Therefore, NEDD4 could suppress PTEN function in a ubiquitination-dependent. Still, the degradation-independent manner in a specific context and further investigations are needed to clarify the molecular mechanism of this regulation mode. As for gastric cancer, NEDD4 was found to be overexpressed in gastric cardia adenocarcinoma, which is closely related to poor prognosis [50]. But NEDD4 expression changes did not correlate with PTEN expression changes during gastric carcinogenesis [51]. In the present study, we also found that the knockdown of NEDD4 has no pronounced effect on the PTEN protein expression. Still, loss of PTEN can reverse the inhibitory effect of NEDD4 knockdown on GC cell proliferation. All these data further confirmed that NEDD4 promotes the proliferation of IGF1/IGF1R abnormally activated GC cells in a PTEN-dependent fashion. As a dose-dependent tumor suppressor, PTEN is an ideal target for a tumor suppressor-reactivated strategy for cancer treatment. E3 ligases that control PTEN’s stability, subcellular localization, and enzymatic activity are potential therapeutic targets for PTEN reactivation [52]. Recently, it has been reported that WWP1 is a novel E3 ligase targeting PTEN, which mediates the non-degradative K27-linked polyubiquitination suppressing the dimerization, membrane recruitment, and function of PTEN [49]. And WWP2 was recently identified as a physiological ubiquitin ligase for PTEN in mice [53]. All this evidence suggests that divergent modifications of PTEN ubiquitination play critical and differential effects on the regulation of PTEN.

Given the heterogeneous characteristics of malignancy, the conception of precision medicine advocates exploring and utilizing a context-dependent therapeutic strategy in cancer treatment. The present study demonstrated that NEDD4 is a potential target in IGF signaling-driven GC. So far, a series of E3 ligases of PTEN has been identified, including NEDD4, WWP1, WWP2 and et al. It is not a striking discovery to confirm that inhibition of an E3 ligase targeting PTEN could have a potential tumor suppressive effect in malignancy. But the most important finding in our study is that the NEDD4 targeted strategy can only efficiently inhibit tumor growth in the IGF1 signaling-driven GC. And the pre-requirement for the tumor suppressive effect of NEDD4 inhibition is the existence of PTEN in GC cells. Furthermore, our gain and loss of functional experiments showed that a PTEN/IRS1 axis is essential for the NEDD4-targeted inhibitory effect on tumor growth in IGF1 signaling-driven GC. Because of the evidence that PTEN functions as a protein phosphatase targeting IRS1 [23], a key mediator to transfer a survival signaling from IGF stimulation to phosphorylated Akt, it can be inferred that PTEN protein phosphatase activation-dependent regulation of a NEDD4/PTEN/IRS1 signaling contributes to the oncogenic role of NEDD4 in IGF1 signaling-driven GC. Besides the lipid phosphatase activity against PI3K/Akt signaling pathway, the protein phosphatase activity of PTEN against oncogenic factors such as the phosphoglycerate kinase 1 and the tyrosine kinase PTK6 also is essential in tumor suppression [26, 54]. As a dual-specific protein and lipid phosphatase, the role of PTEN in tumors is more powerful and complicated. Ubiquitination is a decisive regulator of PTEN, which finely controls the protein stability, enzymatic activity, and subcellular localization of this dominant tumor suppressor in multiple cancers. All identified E3 ligases targeting PTEN have an enzymatic HECT domain, which plays critical roles in tumor progression by regulating PTEN ubiquitination. Interestingly, I3C, a natural component from cruciferous vegetables, displays potent antitumor effects by blocking E3 ligase-PTEN interaction [48, 49]. These results suggest that targeting the HECT domain containing E3 ligases may be a potential therapeutic strategy to restore PTEN tumor-suppressive activity. It promotes the development of therapeutic strategies on protein ubiquitination, which may be a “leverage” in tumor cells to give access to excellent cancer treatment outcomes. And we speculated that the significant effect of NEDD4-mediated PTEN ubiquitination is not to promote PTEN protein degradation but to inhibit its protein phosphatase activation, especially on the substrates involved in the IGF1 signaling pathway. This may reasonably explain what we observed in this study.

## Materials and methods

### Cell lines and drugs

For cell lines, human GC cell lines, BGC803, MKN45, SGC7901, and MKN28, were cultured in RPMI-1640 medium (GIBCO BRL, Grand Island, NY, USA) supplemented with 10% fetal bovine serum (GIBCO BRL) and 100 mg/ml penicillin-streptomycin. Human GC cell line AGS was cultured in DMEM (GIBCO BRL) supplemented with 10% fetal bovine serum (GIBCO BRL) and 100 mg/ml penicillin-streptomycin. HEK293T cells were cultured in DMEM (GIBCO BRL) supplemented with 10% fetal bovine serum (GIBCO BRL) and 100 mg/ml penicillin-streptomycin. Cells were incubated at 37 °C in 5% CO2. Human GC cell lines BGC803, MKN45, SGC7901, MKN28, and AGS, were purchased from Genechem (Shanghai, China). HEK293T cells were purchased from ATCC (Manassas, VA, USA). For drugs, OSI906 and Doxycycline were purchased from Sellechem catalog #S1091 and #S4163, respectively, and the IC50 data was obtained from the Selleckchem website (Selleckchem.com). Heclin was purchased from Sigma-Aldrich (SML1396).

### Protein extraction and Western blotting

For protein extraction, cells were washed with phosphate-buffered saline (PBS) three times and then collected and lysed with RIPA lysis buffer with the phosphatase inhibitor cocktail 3 (Sigma-Aldrich, P0044) at a ratio of 100:1 (v/v). The protein quantification was determined by the BCA reagent kit. For western blotting, an equal amount of denatured protein was fractionated by SDS-PAGE and then transferred to nitrocellulose membranes. After blocking with 5% nonfat milk, the membranes were incubated with the following primary antibodies: IRS1(Cell Signaling Technology; 2390 S; 1:1000), phospho-Akt t308 (Cell Signaling Technology, 2965 S; 1:1000), phospho-Akt s473 (Cell Signaling Technology, 4060 S; 1:1000), Akt (Cell Signaling Technology; 9272; 1:1000), NEDD4-1 (Santa Cruz Biotechnology; H-135; 1:200), PTEN (Cell Signaling Technology; 9188 S; 1:1000), IGF1R (Cell Signaling Technology; 3027 S; 1:1 000), phospho-IRS1 Y612 (Invitrogen; 44-816 G; 1:1000), N-Cadherin (Cell Signaling Technology; 13116 S; 1:1000), Slug (Cell Signaling Technology; 9585 S; 1:1000), Vimentin (Cell Signaling Technology; 5741 S; 1:1000), Snail (Cell Signaling Technology; 3879 S; 1:1000), and β-actin (Sigma-Aldrich; A5316; 1:2000) at 4 °C overnight. After washing with 1× TBST three times, the blots were further incubated with HRP-labeled secondary antibodies. Finally, the target proteins were visualized using the enhanced ECL chemiluminescence method and Tanon 5500 (Tanon Science & Technology; Shanghai, China). Each independent experiment was repeated at least three times.

### Cell viability assay

To detect the distinct responses of GC cells to IGF1R inhibitor, cells were counted and seeded in 96-well plates with 2 × 10^3^ cells per well. After attachment, cells were treated with 2 μg/ml OSI906 (Selleck, S1091) for 0, 12, 24, 36, and 48 h respectively, and treated with 0, 1, 2, 4, and 8 μg/ml OSI906 for 48 h respectively. There were five replicates for every treatment. Then, cells were incubated in 100 μl culture medium containing 10 μl Cell Counting Kit-8 (0.5 mg/ml) reagent for 1–4 h at 37 °C. The absorbance at 450 nanometers (nm) was detected by Bio-RAD (Hercules, CA, USA) Microplate Reader. Then cell viability (%) was calculated. For the detection of cell proliferation, cell Counting Kit-8 assays were performed every day for the following 5 days. There were three replicates for every treatment. The cell proliferation curves were created by Graphpad Prism 5 using the OD value obtained at each time point.

### Lentiviral-mediated shRNA vector and infection

The human IRS1-shRNA-1, human IRS1-shRNA-2, human NEDD4-shRNA-1, and human NEDD4-shRNA-2 were contracted into pLKO.1-TRC cloning vector (Addgene, Cambridge, MA, USA; 10878). The sequences of shRNAs were designed from Sigma-Aldrich (St. Louis, MO, USA; Sigma-Aldrich TRC numbers are as follows: IRS1-sh1: TRCN0000231149; IRS1-sh2: TRCN0000231150; NEDD4-sh1: TRCN0000272477; NEDD4-sh2: TRCN0000272425). HEK293T cells were used to generate lentivirus. When the confluence reaches 60–80%, HEK293T cells were transfected with a mixture of shRNA plasmid, packaging plasmid psPAX2 (Addgene, 12260), and envelope plasmid pMD2.G (Addgene, 12259) in the mass ratio of 4:3:1. Then the virus was harvested 48 and 72 h later and then centrifuged at 1000 revolutions per minute (rpm) for 10 min and purified using a 0.45 micrometer (μm) syringe filter. To generate IRS1/NEDD4 stable-knockdown cell lines, human GC cell lines BGC803, MKN-45 and AGS were plated into six-well plates and incubated with lentivirus and cultured medium supplemented with polybrene (8 μg/ml) when confluence reached 30–50%. 24 h later, cells were treated with 2 μg/ml puromycin for 3 days when there were no survival cells in the blank treatment groups. For the Tet-On expression system, cells were treated with 100 ng/ml Doxycycline (Selleck, S4163) for 48 h.

### Plate clone formation assay

After trypsinized into a single cell suspension, cells were seeded in a 6-well plate at 10^3^ cells per well and cultivated for 2 weeks. After three washes with PBS, cells were fixed with 4% paraformaldehyde for 15 min and stained with 0.5% (w/v) crystal violet dye (Sigma-Aldrich) for 15 min. There were three replicates for every treatment. Images of cell clones were captured using Odyssey Scanner (LI-COR, Lincoln, NE, USA) and counted using ImageJ software.

### The soft agar clone formation assay

Cells were suspended in media containing 0.3% low-melting agarose and then plated on a bottom layer of 0.5% agarose-containing media in a 6-well plate (3000–5000 cells per well). After 2 weeks of incubation, phase-contrast microscopic images were photographed using a digital camera coupled to a microscope, and clones were counted using ImageJ software. There were three replicates for every treatment.

### 5-Ethynyl-20-deoxyuridine (EdU) incorporation and Click-iT ™ reaction

For EdU incorporation experiments, cells were seeded in a 96-well plate at 10^4^ cells per well and treated with EdU (from YF 488 Click-iT™ EdU Imaging Kit, US Everbright, China) at a 50 μM final concentration for 4 h. For the click-it reaction, cells were fixed with 50 μl of 4% paraformaldehyde for 15–30 min, and then 50 μl 2% glycine in deionized water (w/v) was used to terminate the fixation. Cells were washed with 100 μl 3% BSA in PBS (w/v) twice and incubated with 100 μl 5% (v/v) Triton-X100 in PBS for 20 min. After washing by 3% BSA in PBS twice, cells were incubated with 100 μl Click-iT reaction buffer at room temperature away from light for 30 min. After washing with 100 μl 3% BSA in PBS twice and PBS once, cells were incubated with 100 μl 1xHoechst 33342 in PBS at room temperature, away from light 15–30 min. After washing with PBS twice, pictures were taken using an inverted fluorescent microscope. There were three replicates for every treatment. All procedures were performed following the manufacturer’s instructions.

### Xenograft nude mice model

BGC803 cells stably expressing NEDD4-shRNA and control scramble shRNA (5 × 10^6^ cells in 100 μl of PBS) were injected subcutaneously into the back of BALB/c (nu/nu) female mice aged 6–8 weeks (five mice per group, randomly grouping design). The +DOX group was fed with water containing 2 mg/ml doxycycline hydrochloride (shyuanye, China), while the –DOX group was fed with normal water. The maximum (L) and minimum (W) length of the tumor were measured using a slide caliper every 3 days to monitor the tumor growth rate, and the tumor volume was calculated according to the formula V = (L × W^2^)/2. The mice were killed 20 days after injection, and tumors were collected and weighed. All procedures were conducted following the “Guiding Principles in the Care and Use of Animals” (China) and were approved by the Laboratory Animal Ethics Committee of the fourth military medical university (IACUC-20171005).

### Immunohistochemistry (IHC)

IHC for the target molecules was performed on single serial sections made from xenograft tumor samples and tissue microarray chips (Department of Pathology, Fourth Military Medical University). The microarray contained 30 human gastric tumor samples and 30 adjacent normal tissue samples. The slides were probed with antibodies of NEDD4 (Sigma-Aldrich; R36446; 1:200), IGF1R (Cell Signaling Technology; 3027 S; 1:100), and p-Akt s473 (Cell Signaling Technology, 4060 S; 1:100). Immunohistochemistry photographs were scanned by Pannoramic (Santa Clara, CA, USA) MIDI and quantified with histochemistry score (H-Score) by Quant center.

### siRNA and plasmid transfection

The PTEN siRNA-1, PTEN siRNA-2, and negative control (NC) were chemically synthesized and purified by Genepharma. The sense strand sequences of PTEN siRNAs were as follows: PTEN siRNA-1: 5′-UGCAGCAAUUCACUGUAAATT -3′; PTEN siRNA-2: 5′-GAGCGUGCAGAUAAUGACATT -3′; negative control: 5′-UUCUCCGAACGUGUCACGUTT -3′. The NEDD4 plasmid was purchased from Sino Biological (HG11437-NY). siRNAs and plasmid were transfected into BGC803, AGS, and MKN45 cells when the cell confluence was 60-80%. Lipofectamine Reagent 2000 (Invitrogen) and 200 micromoles per liter (μM) of specific siRNAs (1:1) and plasmid were premixed in the serum-free medium and incubated for 20 min at room temperature. After incubation with a Lipofectamine/siRNA or Lipofectamine/plasmid mixture for 6 h, cells were replaced with a fresh normal cell culture medium.

### Clinical data analysis

The mRNA expression of IGF1 and NEDD4 in GC patients was downloaded from Gene Expression Omnibus (GEO, https://www.ncbi.nlm.nih.gov/geo/) and normalized using GEO2R. The correlation of IGF1, IRS1, and NEDD4 expression with the clinicopathologic features of GC patients were analyzed using the assistant for clinical bioinformatics (https://www.aclbi.com/static/index.html#/). The survival analysis of GC patients was downloaded from Kaplan–Meier Plotter (http://kmplot.com/analysis), and we chose the auto-select best cutoff value. We used the probe set 209540_at, 203627_at, 204686_a, and 213012_at to analyze the correlation between the expressions of IGF1, IGF1R, IRS1, and NEDD4 and OS, FP, and PPS of GC patients. The correlation of NEDD4 expression and the IGF1/IGF1R/IRS1 axis was analyzed using the TCGA dataset (https://tcga-data.nci.nih.gov/tcga/). Raw counts of RNA-sequencing data (level 3) and corresponding clinical information from GC were obtained from The Cancer Genome Atlas (TCGA) dataset (https://portal.gdc.cancer.gov/), in which the method of acquisition and application complied with the guidelines and policies. *P* values were based on Spearman’s coefficient test. A *P* value of <0.05 was considered statistically significant.

### Statistical analysis

The in vitro experiments were repeated at least three times. All quantitative data are presented as the mean ± SD of three biologically independent experiments or samples. Statistical analysis was performed using GraphPad Prism 8. Statistical significance was tested using a two-tailed unpaired or paired Student’s *t* test. The analysis of IGF1R expression in GC stratified by N-cadherin expression and normal tissues, NEDD4 expression in GC stratified by N-cadherin expression and normal tissues, and NEDD4 expression in GC stratified by IGF1 expression and normal tissues in TCGA database were analyzed by kruskal-wallis test. The correlation of protein expression of IGF1R, NEDD4 and p-Akt s473 was analyzed by linear regression analysis. The Kaplan–Meier survival was analyzed by Log-rank (Mantel-Cox) test analysis. *p* < 0.05 was considered statistically significant (*p < 0.05, **p < 0.01, ***p < 0.001).

## Supplementary information

supplementary material

## Data Availability

All data needed to evaluate the conclusions in the paper are present in the paper and the supplementary materials. The data supporting this study’s findings are available from the corresponding author upon reasonable request.
