# Supplementary material for: Targeting the E3 ligase NEDD4 as a novel therapeutic strategy for IGF1 signal pathway-driven gastric cancer
Source: Oncogene. 2023 Feb 11;42(14):1072–87. doi: 10.1038/s41388-023-02619-4 (PMC10063445; doi:10.1038/s41388-023-02619-4)
Supplement: Supplementary file 1 — supplementary material [file 41388_2023_2619_MOESM1_ESM.docx]

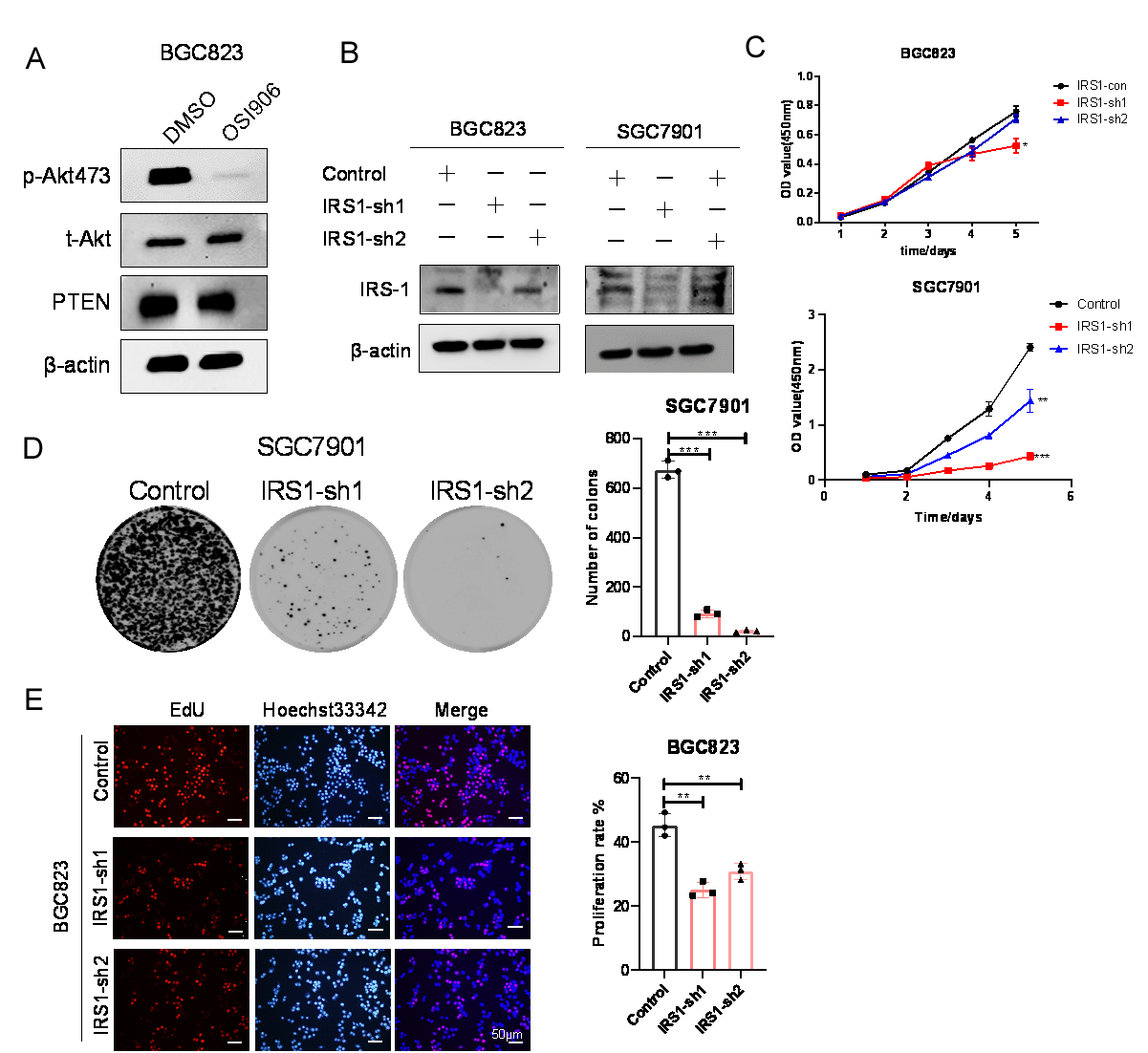


**Figure. S1. An IRS1-PTEN axis is required for proliferation of IGF1R-dependent GC cells.** **A** Western blot analysis of p-Akt s473, t-Akt, PTEN, and β-actin expression in BGC823 treated with DMSO and OSI906 (2μg/ml, 48h). **B** Western blot analysis of IRS1 in the IRS1 stable knockdown cell lines BGC823 and SGC7901. **C** Cell viability detection of BGC823 and SGC7901 cells after IRS1 knockdown by CCK-8 assay. Cell proliferative capacity detection of BGC823 and SGC7901 after IRS1 knockdown by **D** plate clone formation assay and **E** EdU assay. ***p < 0.001, **p < 0.01, *p < 0.05.


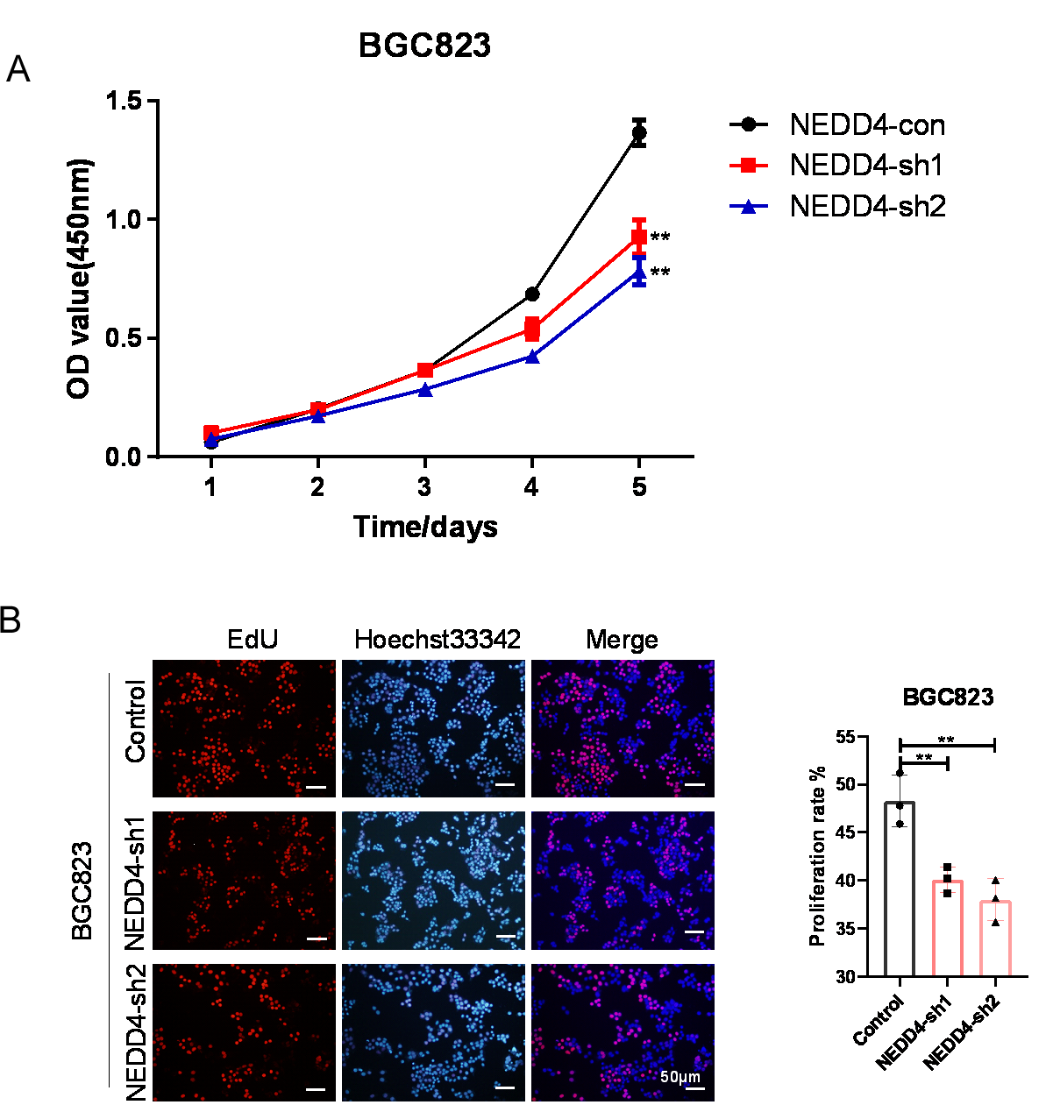


**Figure. S2 NEDD4 promotes proliferation of IGF1R-dependent GC cells.** Cell viability detection of BGC823 cells after NEDD4 knockdown by CCK-8 assay (**A**) and EdU assay (**B**). ***p < 0.001, **p < 0.01, *p < 0.05, ns >0.05.

**Table S1** Correlations between NEDD4 expression and clinical characteristics in patients with GC.

| Variables | Expression of NEDD4 |  | p-value |
| --- | --- | --- | --- |
|  | High (n = 188) | Low (n = 187) |  |
| **Status**  Alive  Dead  **Age (years)** | 104(55.32%)  84(44.68) | 124(66.31%)  63(33.69%) | 0.038*  0.706 |
| Age Mean (SD) | 65.6 (9.9) | 66 (11.4) |  |
| Age Median [Min Max] | 67 [35 90] | 68 [39 90] |  |
| **Gender** |  |  | 0.564 |
| Male | 124 (65.96%) | 117 (62.57%) |  |
| Female | 64 (34.06%) | 70 (37.43%) |  |
| **Grade of differentiation** |  |  | 0.382 |
| G1 | 5 (2.66%) | 5 (2.67%) |  |
| G2 | 70 (37.23%) | 67 (35.83%) |  |
| G3 | 106 (56.38%) | 113 (60.43%) |  |
| GX | 7 (3.72%) | 2 (1.07%) |  |
| **Tumor invasion** |  |  | 0.061 |
| T1 | 6 (3.91%) | 13 (6.95%) |  |
| T2 | 35 (18.62%) | 45 (24.06%) |  |
| T3 | 78 (41.49%) | 90 (52.94%) |  |
| T4 | 62 (32.98%) | 38 (20.32%) |  |
| TX | 7 (3.72%) | 1 (0.53%) |  |
| **Lymph node status** |  |  | 0.216 |
| N0 | 48 (25.67%) | 63 (33.87%) |  |
| N1 | 50 (26.74%) | 47 (25.27%) |  |
| N2 | 37 (19.79%) | 38 (20.43%) |  |
| N3 | 40 (21.39%) | 34 (18.28%) |  |
| NX | 12 (6.42%) | 4 (2.1%) |  |
| **Distant metastasis** |  |  | 0.982 |
| M0 | 165 (87.77%) | 165 (88.23%) |  |
| M1 | 13 (6.91%) | 12 (6.42%) |  |
| MX | 10 (5.32%) | 10 (5.35%) |  |
| **TNM stages** |  |  | 0.287 |
| I | 24 (13.33%) | 29 (16.38%) |  |
| II | 50 (27.78%) | 61 (34.46%) |  |
| III | 83 (46.11%) | 72 (40.68%) |  |
| IV | 23 (12.78%) | 15 (8.47%) |  |

*Statistically significant. P-value < 0.05 are in bold.


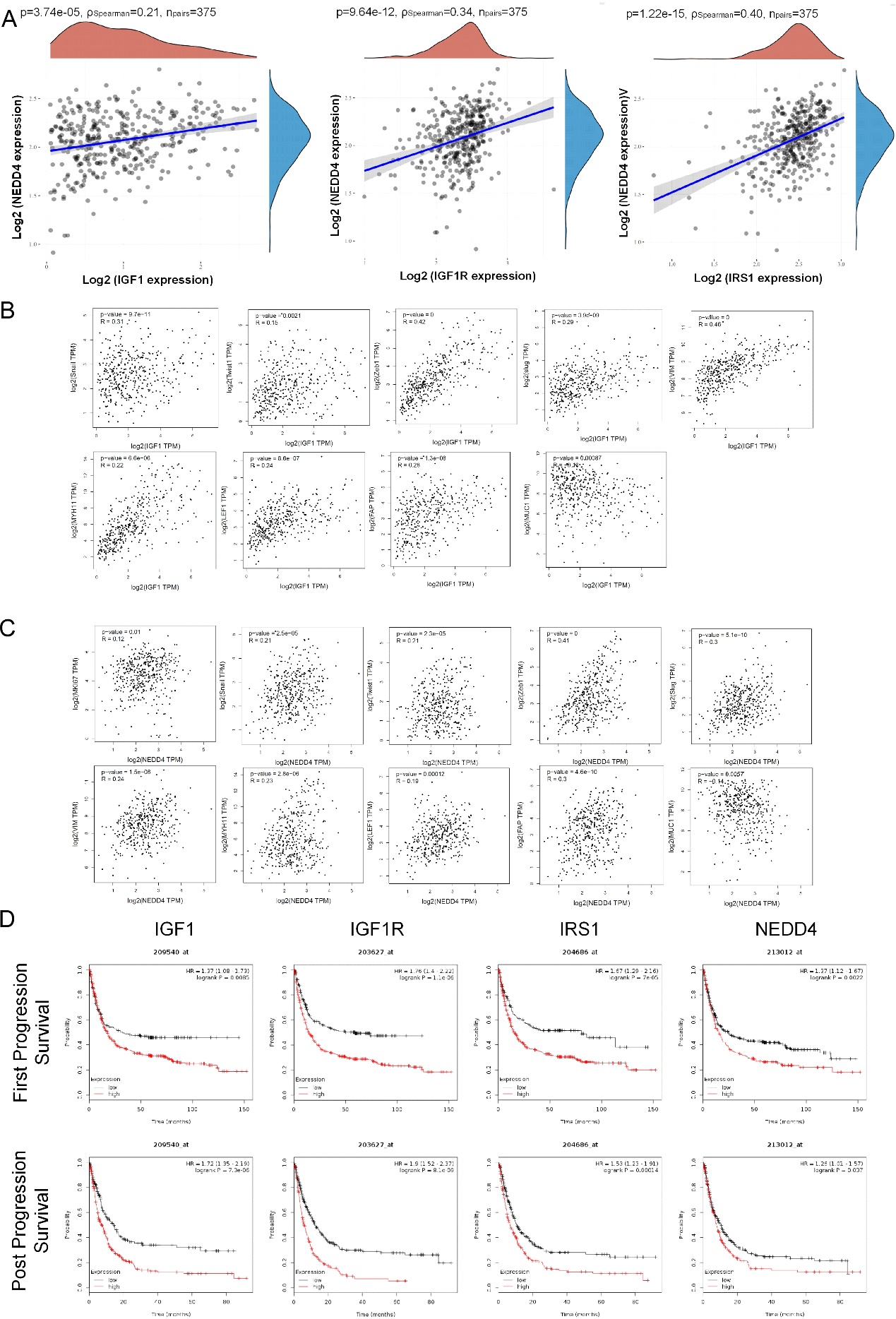


**Figure. S3 The prognostic value of the IGF1-R/NEDD4/IRS1/PTEN/AKT axis in GC.** **A** Correlation of NEDD4 with the IGF1/IGF1R/IRS1 axis in GC in TCGA dataset. **B** Correlation of IGF1 with mesenchymal subtype markers (including Snail, Twist1, Zeb1, Slug, VIM, MYH11, LEF1, and FAP), and epithelial subtype marker MUC1 in GC. **C** Correlation of NEDD4 with tumor proliferation marker Ki67, mesenchymal subtype markers (including Snail, Twist1, Zeb1, Slug, VIM, MYH11, LEF1, and FAP), and epithelial subtype marker MUC1 in GC. **D** Kaplan–Meier analysis of the correlation between IGF1, IGF1R, IRS1, and NEDD4 expressions and FP and PPS of GC patients. ***p < 0.001, **p < 0.01, *p < 0.05, ns p>0.05.
